# DNA under Force: Mechanics, Electrostatics, and Hydration

**DOI:** 10.3390/nano5010246

**Published:** 2015-02-25

**Authors:** Jingqiang Li, Sithara S. Wijeratne, Xiangyun Qiu, Ching-Hwa Kiang

**Affiliations:** 1 Department of Physics and Astronomy, Rice University, Houston, TX 77005, USA; E-Mails: jingqiang.li@rice.edu (J.L.); sitara@rice.edu (S.S.W.); 2 Department of Physics, George Washington University, Washington, DC 20052, USA; 3 Department of Bioengineering, Rice University, Houston, TX 77005, USA

**Keywords:** DNA, mechanics, electrostatics, hydration

## Abstract

Quantifying the basic intra- and inter-molecular forces of DNA has helped us to better understand and further predict the behavior of DNA. Single molecule technique elucidates the mechanics of DNA under applied external forces, sometimes under extreme forces. On the other hand, ensemble studies of DNA molecular force allow us to extend our understanding of DNA molecules under other forces such as electrostatic and hydration forces. Using a variety of techniques, we can have a comprehensive understanding of DNA molecular forces, which is crucial in unraveling the complex DNA functions in living cells as well as in designing a system that utilizes the unique properties of DNA in nanotechnology.

## 1. Introduction

With the advances in nanomanipulation techniques, researchers are able to directly manipulate and measure the molecular force of DNA at the single molecule level, which expands our understanding of DNA mechanics [1,2,3]. With DNA being a long and thin chain of nucleotides, single molecule techniques are uniquely advantageous in pulling on this linear chain to allow us to quantify the forces and fit the data to theoretical models in order to understand its behavior. On the other hand, molecular forces involved in compacting DNA into high densities have been traditionally studied via ensemble approaches, e.g., pushing DNA strands together by simply concentrating DNA solution [4] or adding crowding osmolytes [5]. Particularly, the ease of condensing DNA via multivalent cations [6], as well as the challenge of its mechanistic elucidation, has spurred broad interests in DNA-inspired electrostatics [7,8] and the roles of hydration in molecular interactions [9]. Single molecule and ensemble studies, acting synergistically, have yielded a comprehensive understanding of the physics of DNA molecular forces that is unlikely to be reproduced for other biomolecules.

## 2. Mechanical Forces in DNA

The conformation of DNA and its resulting mechanical properties are crucial in a variety of biological processes, such as replication, transcription, gene regulation, and genome compaction. The intra- and inter-molecular forces of DNA play a significant role in the operation of cellular machinery, including how it wraps around histones, packs into phage heads, and interacts with proteins. The potential of using DNA in nanotechnology has been explored due to its favorable characteristics, such as its capability of programmable self-assembly and resulting stiffness due to base-pairing [10,11,12]. For example, DNA can be used as a building block for complex nanostructures as well as performing computation [11,13,14,15,16,17]. Over the last two decades, with well-developed piconewton instrumentation, researchers were able to directly measure the molecular force of DNA at the single molecule level, which incredibly expands our understanding of DNA mechanics.

**Figure 1:**
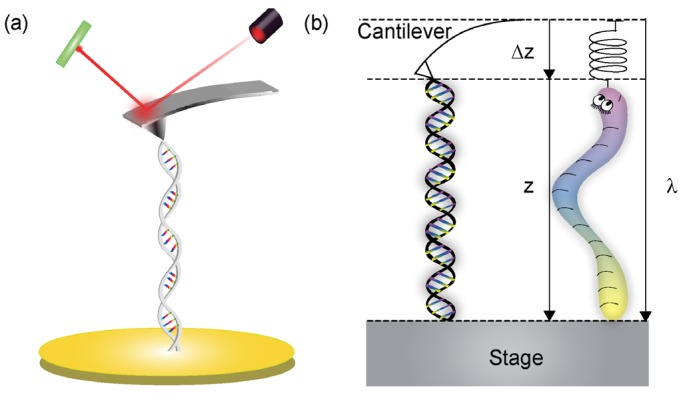
Illustration of a single molecule manipulation experiment using atomic force microscopy (AFM). (**a**) One end of the DNA molecule is attached to a substrate while the other end is pulled by the AFM cantilever tip; (**b**) The cantilever spring obeys Hooke’s law and the elasticity of DNA follows the wormlike chain (WLC) model. The stage position, λ, is related to the molecular end-to-end distance, *z*, by *z* = λ – Δ*z*. Adapted from [33].

Both double-stranded DNA (dsDNA) and single-stranded DNA (ssDNA) may be viewed as a polymer. Single molecule manipulation has been used extensively to study the force response of a biopolymer [1,18,19,20,21,22] (Figure 1). Examples of several widely used techniques, which can measure piconewton level forces, are atomic force microscopy (AFM) [2], optical tweezers [1,23], magnetic tweezers [24], glass microneedles [25], and biomembrane force probes [26]. These techniques involve stretching a single molecule while monitoring its force response. They have been used to characterize the mechanical properties and the forces associated with the conformational changes of ssDNA and dsDNA molecules. Melting transitions were observed by repeatedly stretching and relaxing double-stranded λ-DNA molecules [27], while the unwinding forces have been determined by torque studies [28,29,30,31,32].

Polymer physics models were successfully used to describe the mechanical behavior of stretched DNA obtained from single molecule force measurement [34]. The elasticity of DNA can be described by using one-dimensional polymer physics models. wormlike chain (WLC) and freely joined chain (FJC) models [35] are usually used to interpret the force-extension curves of DNA stretching. The FJC model assumes a polymer chain consisting of *n* segments of characteristic length $l_{k}$ (Kuhn length), connected via freely-rotating joints with contour length $l_{c}=nl_{k}$:

(1) $$z\left(F\right)=l_{c}\left[\text{coth}\left(\frac{Fl_{k}}{k_{B}T}\right)-\frac{k_{B}T}{Fl_{k}}\right]$$

where F is the force; $k_{B}$ is the Boltzmann constant; and T is the temperature. Stretching ssDNA and dsDNA can be described by extensible FJC (eFJC) model [23]:

(2) $$z\left(F\right)=l_{c}\left[\text{coth}\left(\frac{Fl_{k}}{k_{B}T}\right)-\frac{k_{B}T}{Fl_{k}}\right]\left(1+\frac{F}{k_{seg}l_{k}}\right)$$

which assumes the additional extension by modeling each segment as an elastic spring with segment elasticity $k_{seg}$. On the other hand, the WLC model treats a polymer molecule as a homogenous elastic rod, or a wormlike chain, characterized by its contour length, $l_{c}$, and persistence length, $l_{p}$, which characterizes the bending stiffness of the WLC:

(3) F(z)=kBTlp[14(1−zlc)2−14+zlc]

For lengths longer than $l_{p}$, it is assumed that the correlation between tangents to the polymer is lost.

The mechanical stretching of dsDNA is best described by the extensible WLC model (eWLC) [36,37]:

(4) $$z\left(F\right)=l_{c}\left[1-\frac{1}{\sqrt{4l_{p}F/k_{B}T}}+\frac{F}{K_{ds}}\right]$$

where $K_{ds}$ is the elastic stretch modulus for dsDNA. In single molecule experiments, for the part of the curve that is below the plateau, the following values are obtained: $l_{p}$ = 50, and $K_{ds}$ = 1200 pN, which are consistent with theory [18,38].

### 2.1. Mechanics of Double-Stranded DNA

Single molecule force experiments have revealed in detail the mechanical properties of dsDNA. These studies have provided significant insights into the intra-molecular force interactions of DNA. The studies on short dsDNA focus on the force-induced melting and the sequence-dependent effects [39,40,41,42]. Those on long dsDNA address its overall properties, particularly elasticity [1,2,18,38,43,44,45,46,47].

#### 2.1.1. Short dsDNA

**Figure 2:**
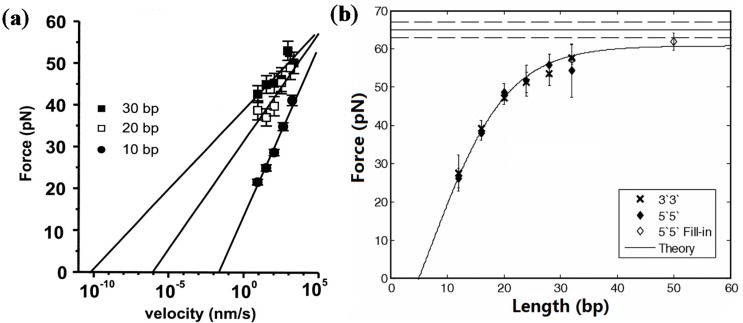
(**a**) Velocity dependence of the rupture force. Data showing the most probable rupture force as a function of stretching velocity for the short dsDNA of 30 bp, 20 bp, and 10 bp. The rupture force scales linearly with the logarithm of velocity. Adapted from [48]. Copyright (1999) National Academy of Sciences, USA; (**b**) Dependence of the rupture force on the length of dsDNA. The central horizontal line is the critical force of the overstretching transition measured in the λ-phage DNA. The upper and lower lines correspond to 10% and 90% of overstretching transition, respectively. Adapted from [39]. Reprinted with permission. Copyright (2005) American Physical Society.

Short dsDNA refers to DNA with a length of fewer than 100 base pairs (bp). To characterize the melting of a short dsDNA, single molecule techniques measure rupture force as a function of pulling velocity (Figure 2a) [48,49]. Kinetic melting information is obtained by fitting the data to the Bell’s model. On the other hand, the rupture force of short dsDNA increases with the number of base pairs. For 12–20 base pairs, the rupture force scales linearly with the DNA length [42]. The rupture force was found to reach a limit of 61 pN, when the base pair number increases over 30 (Figure 2b) [39].

#### 2.1.2. Long dsDNA

**Figure 3:**
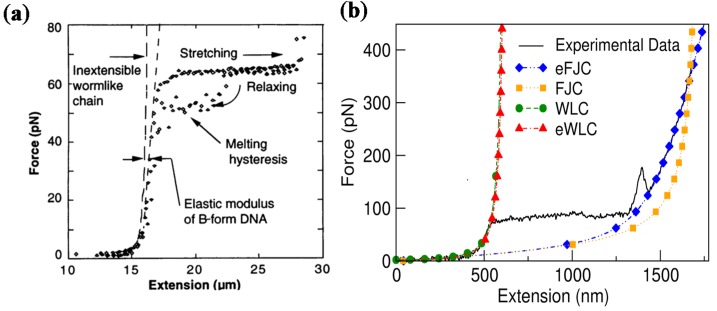
Experimental force-extension data for the stretching of λ-phage DNA. (**a**) The data are obtained from optical tweezers and fitted with the WLC model. Adapted from [1]. Reprinted with permission from AAAS; (**b**) Data are obtained from AFM single molecule experiments and fitted with different one-dimensional polymer models. Adapted from [33]. Reprinted with permission from Springer Science and Business Media.

A typical force-extension curve of λ-phage DNA with a length distribution of 117–8454 bp obtained from a single molecule force experiment is shown in Figure 3. It exhibits three states of the double helix during the stretching. In the first regime, the force is extremely small, and the DNA is in its B-form. Fitting the force-extension curve to the WLC model gives a persistence length of 53 nm (Figure 3a) [1] and the extensible WLC model gives a persistence length of 50 nm and a stretch modulus of 1200 pN (Figure 3b) [27]. Continuing stretching, DNA undergoes a B-S transition to its overstretching state indicated by the constant force regime at 65 pN. The length stretches up to 1.7 times its B-form length and the DNA conformation changes to the S-form [1,50,51,52,53,54,55]. After the overstretching transition, DNA transitions into single strands or possibly melts at forces of 150 pN. The extensible FJC is used to fit the high force regime, and the resulting persistence length and stretch modulus are consistent with that of ssDNA.

### 2.2. Mechanics of Single-Stranded DNA

**Figure 4:**
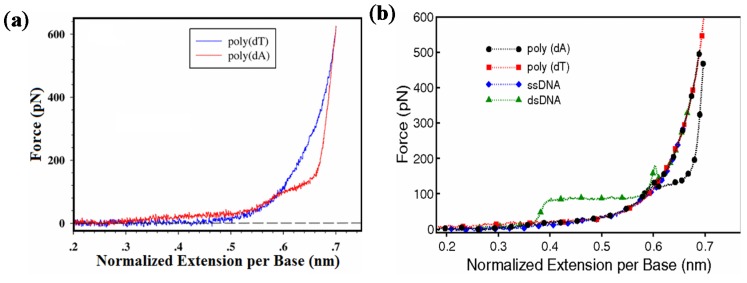
Force-extension curves for ssDNA. (**a**) Comparison of pulling curves between poly(dT) and poly(dA). Reprinted with permission from [60]. Copyright (2007) by the American Physical Society; (**b**) Force-extension curves for different forms of dsDNA and ssDNA. Reprinted with permission from [3]. Copyright (2010) by the American Physical Society.

Single-stranded DNA does not have the base pairing interaction found in dsDNA. However, some ssDNA shows base stacking interaction, which can significantly affect its elasticity and conformation. Among the four bases of DNA, base stacking is strongest among adenine (A) bases and weakest among thymine (T) bases [56,57]. Figure 4 is a typical force-extension curve of poly(dA) and poly(dT), showing the two plateaus in the force-extension curve of poly(dA). The first plateau begins at 23 pN and overstretches about 75% of its original stacked state length, which agrees well with the prediction of a theoretical model [58,59,60]. Poly(dA) force curves show multiple plateaus and multiple pathways when stretched, whereas poly(dT) force curves can be fitted with a simple FJC model. This suggests the existence of complex intra-molecular interactions in poly(dA). The existence of A-tract in DNA may be related to its unique mechanical properties [61].

## 3. Electrostatic and Hydration Forces in DNA

**Figure 5:**
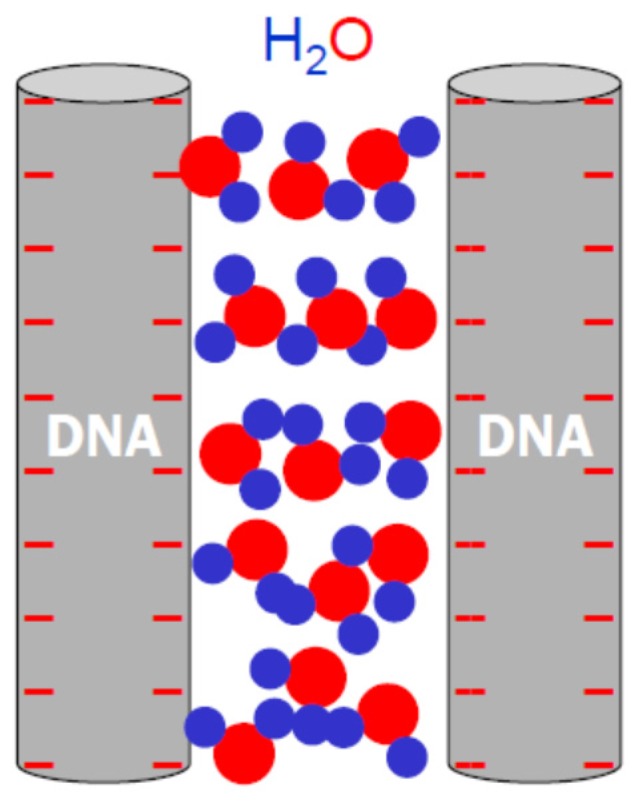
A cartoon illustration of DNA–DNA interactions in a side-by-side configuration.

Consider two parallel dsDNA helices approaching each other from a distance in solution, as illustrated in Figure 5; the force between them as a function of inter-axial distance defines the analytical form and physical origin of DNA–DNA interactions. At inter-axial distances greater than 3 nm (*i.e.*, ≥1 nm surface separation), DNA–DNA interactions are dominated by long-range electrostatic forces due to the highly charged nature of DNA. The dependence on the relative azimuthal angle of the two helices is minimal, and each DNA is free to rotate along its cylindrical axis. Within the final nanometer of surface separation, overlapping of the respective hydration shell of opposing DNA helices leads to (de)hydration forces. In addition, the dielectric discontinuity between DNA and its surroundings results in fluctuation-induced van der Waals interactions, which, however, are comparatively rather weak and not discussed here [62]. As the DNA helices (e.g., less than 0.4 nm surface separation) approach each other, DNA–DNA interactions show significant dependence on the relative azimuthal angle as the “ruggedness” of DNA surfaces becomes relevant [4,63,64]. The strong force at such short distances strongly disrupts the hydration shells and is able to induce conformational changes of DNA [65]. Therefore, a complete and quantitative description of DNA–DNA force–distance relationships necessitates physical understanding of all pertinent components of the system: ions, solvent (water), and helical DNA. While it is largely true that researchers have reached consensus on the fundamental physical interactions at play (e.g., electrostatics, hydration, charged surfaces), quantitative accounts of these interactions are yet to be realized.

With the goal of quantitative understanding of DNA–DNA interactions (and nucleic acid interactions in general), extensive studies, both experimental and theoretical, have been carried out in the past decades. These efforts have resulted in a large body of experimental observations and insightful theoretical models that have substantially improved our knowledge of the behavior of highly charged biomolecules in ionic solutions. Due to the limited scope of this review, readers are referred to many excellent review articles on nucleic acid interactions or polyelectrolyte behaviors for in-depth discussions (e.g., a few recent review articles, as in [7,66,67,68,69,70,71,72,73,74]). In what follows below, rather than being exhaustive, we aim to provide a general overview of relevant physical interactions at play and to promote productive thinking and discussion of the contentious issues in our current understanding.

Before getting started, it is useful to define several important quantities that convert familiar experimental conditions to relevant physical parameters:

(1) Bjerrum length ${\text{λ}}_{B}=\frac{e^{2}}{4{\text{πεε}}_{0}k_{B}T}$, where *e* is the electron charge, ε is the dielectric constant of the solvent (e.g., 78.3 for water at 25 °C), and *k_B_T* is the thermal energy. Mathematically, when two ions of elemental charge are separated by Bjerrum length, the electric potential energy is the same as the thermal energy, giving a measure of the length scale of ion–ion correlations which is about 0.7 nm in water at 25 °C. It is worth noting that for ions of higher valence, the effective Bjerrum length is reduced by a factor of the product of the valences of the ion pairs.
(2) Ionic strength $I=\frac{1}{2}\sum_{i}z_{i}^{2}n_{i}$, where the sum runs over all ionic species; z*_i_* is the valence of ion; *i*, and *n_i_* is the concentration of ion *i.* To put the role of ionic valence in perspective, 100 mM NaCl and MgCl_2_ solutions will give an ionic strength of 100 mM and 300 mM, respectively. Without resorting to any physical formalism, ionic strength can be considered as an empirical way to characterize the effect of ion screening.
(3) Screening Debye length ${\text{λ}}_{D}=\sqrt{\frac{{\text{εε}}_{0}k_{B}T}{\sum_{i}z_{i}^{2}e^{2}n_{i}}}=\sqrt{\frac{1}{4{\text{πλ}}_{B}\sum_{i}z_{i}^{2}n_{i}}}=\sqrt{\frac{1}{8{\text{πλ}}_{B}I}}$. Debye length thus considers the roles of both thermal motion and ion screening. Mathematically, it is the decay length of the electrostatic field in ionic solutions that takes on an exponential form rather than a power law in dielectric medium as a result of ionic screening. For example, λ*_D_* is ~1 nm at 100 mM monovalent salt. However, it should be noted that Debye length is strictly speaking only applicable to weak electrostatic fields, as it is derived from a linearized version of the Poisson-Boltzmann (PB) mean-field treatment of electrostatic interactions.
(4) Charge densities of charged biomolecules, linear charge density η, and surface charge density σ. In the case of dsDNA of 1 nm radius and 2 e per 0.34 nm, η is 5.88 e/nm and σ is 0.94 e/nm^2^.

### 3.1. The Role of Ions

Without ions, electrostatic repulsion between DNA helices would dominate DNA–DNA interactions. An instructive example is given by the energetics of DNA packaging in bacterial viruses. Taking the case of λ phage with 48,502 bp dsDNA and 30 nm radius, a simple model calculation based on a uniformly charged sphere gives 5 × 10^8^ pN·nm electric potential energy if stored in water alone, which is thousands of times greater than the measured work during DNA packaging [75,76]. It is not surprising that these bacterial viruses will burst open due to the strong internal DNA forces at low salt. The presence of ions in physiological conditions can therefore reduce the electrostatic penalties by several orders of magnitude! In addition to the role of screening DNA–DNA repulsive forces, multivalent ions of valence ≥3 are able to induce attractive forces between DNAs at sub-millimolar concentrations, giving rise to the phenomenon of DNA condensation [77]. Such delicate dependence on ionic valence has put ions at the center of studies of DNA-inspired electrostatics. We would like to note that this review focuses on the ions that are generally considered to non-specifically interact with DNA, e.g., Na^+^, K^+^, Mg^2+^; conversely, a wide variety of metal ions can associate with and condense DNA through specific bindings that often distort or completely disrupt the double helical structure [78].

#### 3.1.1. Screening of DNA–DNA repulsion

**Figure 6:**
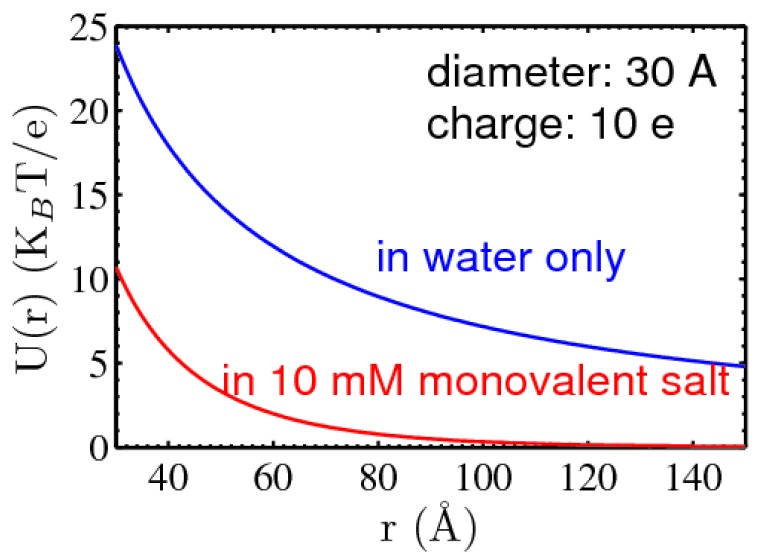
Visualization of Debye–Hückel potentials between two charged spheres of 3 nm diameter and 10 e bare charge. The effect of salt screening is shown for the case of 10 mM monovalent salt.

The seminal DLVO theory (named after Derjaguin, Landau, Verwey, and Overbeek [79]), originally developed to describe the behavior of charged colloids, provides a convenient starting point to quantitate the electrostatic repulsion between DNA. The potential energy between charged molecules is given as the Debye-Hückel (DH) potential $U\left(r\right)=\{\begin{array}{c} \infty \;,\;r<D\\ \frac{Z^{2}}{\text{ε}{\left(1+\text{κ}D/2\right)}^{2}}\frac{e^{-\left(r-D\right)}}{r},\;r\geq D \end{array}$, where *D* is the diameter of the molecule, κ is the inverse Debye length; and *Z* is the molecular charge. Figure 6 illustrates the shapes of DH potentials for two charged spheres in pure water and 10 mM monovalent salt. While the analytical form above is only true for two charged spheres, adapting it to cylindrical charge, non-trivial analytically [80], can be carried out numerically or by treating DNA as spherical beads on a chain. However, the more deeply rooted problem is that the DLVO theory is based on a linearized PB equation. In the PB approach, describing the thermodynamic properties of ions in an electric field, the role of ion-mixing entropy is captured by the Boltzmann factor; it is a mean field approach as the ions are treated as continuous charge densities or spatial distribution probabilities ρ(*r*) as below:

(5) $$\nabla^{2}\text{φ}\left(r\right)=-\frac{4\text{π}}{\text{ε}}\sum_{i}{\text{ρ}}_{i}\left(r\right)=-\frac{4\text{π}}{\varepsilon }\sum_{i}{\text{ρ}}_{i}\left(\infty \right)e^{-\text{β}Z_{i}\text{φ}\left(r\right)}$$

where φ(*r*) is the electric potential and β is 1/k_B_*T*. With appropriate boundary conditions, such as the dimension and charge density of DNA and the ion concentrations at infinity ρ_*i*_(*r*), the non-linear PB (NLPB) equation can be solved and give a complete description of the electric field and ion distribution in the space. The NLPB equation is linearized in the DLVO theory due to the relatively weakly charged nature of the colloids in consideration and the convenience of an analytical solution by taking $e^{-\text{β}z_{i}\text{φ}\left(r\right)}\sim 1-\text{β}z_{i}\text{φ}\left(r\right)$. However, this is no longer justified for highly charged DNA where the $\text{β}z_{i}\text{φ}\left(r\right)$ is not small but even larger than 1 in its vicinity. A linear PB (LPB) equation consequently underestimates the extent of ionic screening, to which many remedial solutions have been proposed.

Counterion condensation, or Manning condensation, represents a significant conceptual advance towards the understanding of electrostatics of highly charged polyelectrolytes such as DNA [81,82]. The criterion for the occurrence of counterion condensation is based on a dimensionless Coulomb coupling strength or the Manning ratio, Γ = λ_B_η: If Γ is greater than 1, *i.e.*, more than one charge is present in a span of Bjerrum length, counterions can condense around the polyelectrolyte to neutralize part of its charge. Then, the LPB approach can be used to describe the electrostatics of the partially neutralized polyelectrolyte. The extent of neutralization depends on the counterion valence z, given as $1-\frac{1}{z\text{Γ}}$. For dsDNA, Γ is 4.1, giving the DNA charge neutralization by mono-, di-, and tri-valent counterions of 76%, 88%, and 92%, respectively. The counterion condensation model is similar in origin to the termed Stern layer in describing the zeta potential of charged colloids. In light of its simplicity and underlying assumptions, the counterion condensation should be viewed only as a first-order approximation of the non-linear screening of counterions. The rich physics of ion atmospheres near polyelectrolyte surfaces has promoted a series of theoretical and experimental studies to achieve quantitative understanding of the non-linearity and its intricate dependence on the ion size and valence and molecular surfaces.

The charge renormalization ansatz took the idea of partial charge neutralization further and sought a thermodynamically more rigorous method to determine the effective charge [83,84,85,86]. Instead of considering a single polyelectrolyte surrounded by ions, it analyzes a cell model of polyelectrolyte dispersions, for example, spherical cells for anisotropic solutions and cylindrical cells for nematically ordered polyelectrolytes. In order to obtain the effective charge to be used in the DH potential, the charge renormalization formalism proposed to match the electrical potential at the cell boundary between LPB (with a so-called renormalized charge) and NLPB (with the bare charge) solutions. Such renormalized charge suited to LPB approaches is expected to yield the same osmotic pressure as the full NLPB treatment. Contrary to the counterion condensation, which predicts the same charge neutralized regardless of polyelectrolyte or ion concentration, the charge renormalization formalism predicts the dependence of renormalized charges on both polyelectrolyte and ion concentrations.

**Figure 7:**
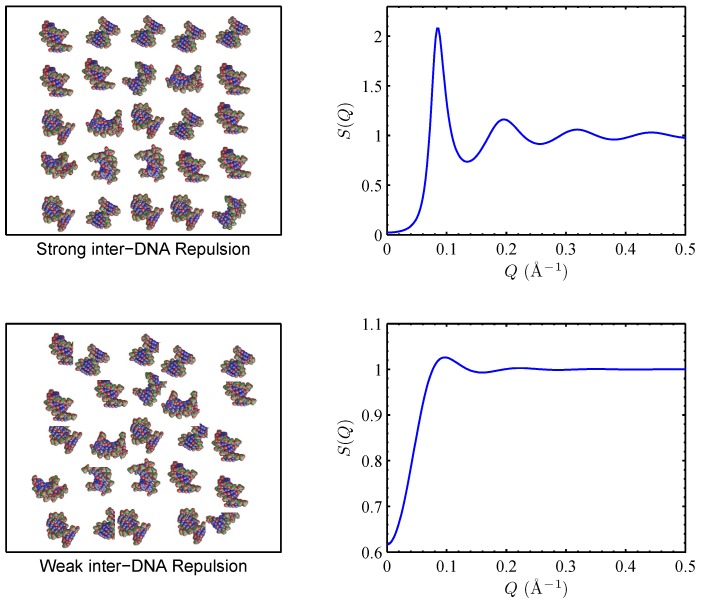
Application of small angle X-ray scattering (SAXS) to measure the structure factor *S*(*Q*) of semi-dilute dispersions of oligomeric DNAs. The top panels show the case of strong DNA–DNA repulsion, giving rise to spatial ordering of DNA strands and a structure factor with pronounced correlation peaks. The bottom panels show the case of weak DNA–DNA interaction, giving rise to random dispersions and a much suppressed structure factor.

**Figure 8:**
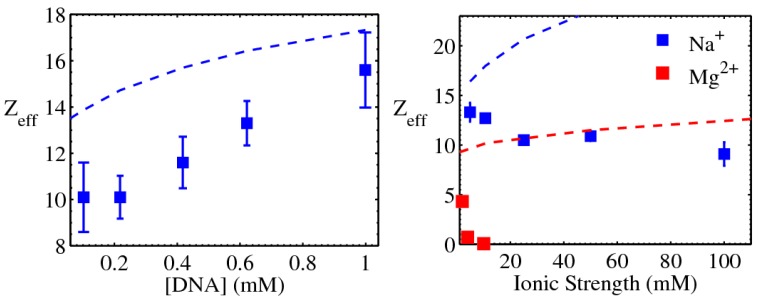
Effective charges (z_eff_) determined by SAXS measurements in conjunction with physical modeling. The oligomeric DNA has a bare charge of 48 e. Symbols are experimental values and lines are the renormalized charges. Details of experimental conditions and modeling procedures are described in [91]. Adapted from [91]. Copyright (2006) by the American Physical Society.

Early experimental guidance and validation for many of these theoretical advances came from the fields of colloidal interactions and general polyelectrolyte behaviors. Here the dispersion of DNA helices provided an ideal physically and biochemically well-defined system for quantitative measurements and modeling of polyelectrolyte interactions. As there is no practical method to hold two DNA helices in space and move them towards each other to accurately measure their forces (note that, while such geometry can be realized experimentally, the forces between two DNA helices at long distances will be too weak for reliable determination), DNA–DNA interactions have been probed by a variety of thermodynamic measurements such as salt-dependent persistence length of dsDNA [87,88] and the osmotic pressure of DNA dispersions [89]. A more recent series of studies employed small angle X-ray scattering (SAXS) to measure the structure factor of dispersions of oligomeric DNAs of uniform sequence and length [90,91]. As demonstrated in Figure 7, the structure factor encodes the spatial correlation of dispersed DNAs, which in turn is determined by the DNA–DNA force-distance relationship, which can be obtained via model fitting. These studies verified that the DH functional form is able to quantitatively reproduce the measured structure factors with one fitting parameter, the effective charge z_eff_. As shown in Figure 8, comparisons with the theoretical renormalized charges showed fairly good agreement given the rather crude nature of the theoretical model, treating dsDNA as uniform cylinders [91]. But significant deviations were observed for even divalent salts [92], which will be discussed later. Overall, the electrostatics of DNA dispersions in monovalent salts has been largely understood.

#### 3.1.2. Mediation of DNA–DNA attraction

Even for divalent ions such as Mg^2+^, our understanding is far from adequate. In the studies of oligomeric DNA dispersions discussed above, large discrepancies exist between theory and experiment in low salt ([Mg^2+^] < 10 mM) when DNA–DNA forces are repulsive (see Figure 8), and, furthermore, DNA–DNA attraction was observed at [Mg^2+^] > 10 mM. While later studies showed that such attraction may be due to end-to-end stacking of the oligomeric dsDNA [93], counterion-mediated attraction between dsDNA helices is undoubtedly established for multivalent cations with valence ≥3, often referred to as “like-charge attraction” or DNA condensation [6,94,95].

**Figure 9:**
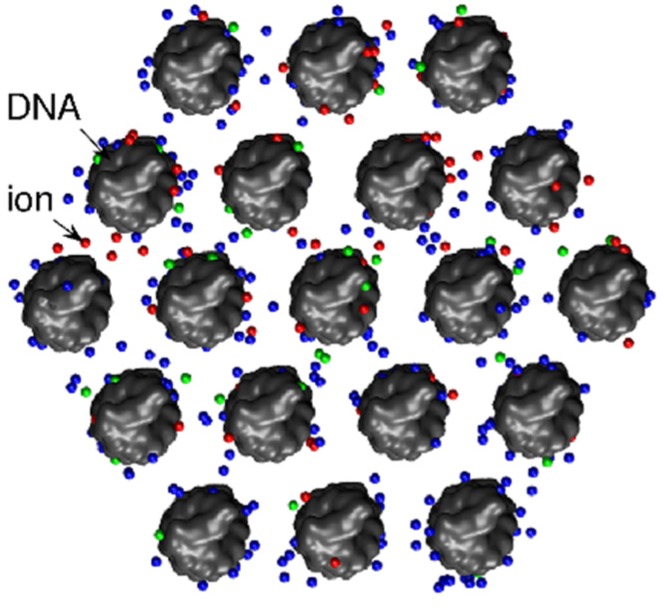
Condensed dsDNA helices packaged in hexagonal arrays, as viewed from the axis. Reproduced from [102]. Copyright (2013) with permission from Elsevier.

The glaring disparity between DNA condensation and DNA–DNA repulsion invariably predicted by mean-field theories has stimulated vast scientific interest and intense studies in the past decades. The most commonly studied DNA-condensing counterions are trivalent Cobalt Hexammine (CoHex), trivalent Spermidine, and tetravalent Spermine [65,96,97,98]. It is known that the condensed DNA helices are packed side by side (*i.e.*, in parallel) in hexagonal arrays, as illustrated in Figure 9, and the as-condensed arrays have inter-axial distances between 2.7 and 3 nm, leaving 0.7–1 nm interstitial space between dsDNA surfaces [99,100,101]. Multivalent cations (and solvent) thus reside in the interstitial space and mediate DNA–DNA attraction. Adding mono- or divalent cations reduces the attraction and eventually redissolves condensed DNA, suggesting an electrostatic-driven attraction. However, the physical origin of attraction is still under debate, as well as the true behavior of ions in such a tight space of extremely high electrostatic fields. To put the composition of the interstitial space in perspective, the effective concentrations of mono-, di-, and trivalent cations, if the only counterion species to neutralize DNA charge, are in the orders of 2000, 1000, and 700 mM, respectively.

It is generally accepted that the mean field description of continuous and static ionic clouds is no longer valid for DNA condensation. One of the early theoretical models involves density fluctuations of these “territorially bound” interstitial counterions, leading to a van der Waals-like attraction [103,104,105]. However, there has been no report of cation-charged density waves in condensed dsDNA arrays. It is worth noting that charge density waves were observed with divalent-cation-condensed F-actin [106], which is, however, complicated by the periodic patches of charges on the F-actin surface. On the other hand, the discrete nature of ions has been considered in a number of competing theoretical models. When DNA is still considered as a uniformly charged cylinder, the “Wigner lattice” model argues for the formation of crystal-like ordering of counterions due to strong ion–ion correlations [107,108,109,110], in a way similar to the ordering of electron gases confined on a positively charged surface. The lattice cohesive energy, as a result of ordering that lowers ion–ion correlation energy, creates correlation holes that lead to attraction between two such lattices. This ion–ion correlation-based model further predicts over-compensation of DNA charge by counterions, *i.e.*, overcharging. DNA overcharging has successfully explained the redissolvation of condensed DNA helices at higher counterion concentrations [111], though there exist alternative explanations involving counterion–coion pairing [101]. Consideration of the discrete nature of charged DNA groups further led to several distinct physical mechanisms for DNA–DNA attraction. The strong Coulomb coupling theory proposes close confinement of counterions and demonstrated net attraction via additive electrostatic interactions of DNA and ions [112,113]. Without taking the strong coupling limit, it has been shown that the undulations of the electrostatic fields due to the molecular nature can result in attraction through optimization of approaching surfaces [114,115]. Moreover, the transient localization of ions near charged groups can result in charge inversion and/or ion bridges. The tightly bound ion model considers such possibility and showed significant DNA–DNA attraction through exhaustive sampling and subsequent NLPB calculations [116,117]. Instead of charge localization near charge groups, the “DNA-ion zipper” model takes into account the DNA major grooves that can accommodate counterions, forming helical spirals of alternating charges [118]. A zipper can thus be constructed between opposing DNA surfaces. The necessitated geometric commensuration has inspired the conjecture and modeling of sequence-dependent DNA–DNA forces, predicting the electrostatics-driven recognition between homologous sequences [119].

### 3.2. The Role of Solvent

**Figure 10:**
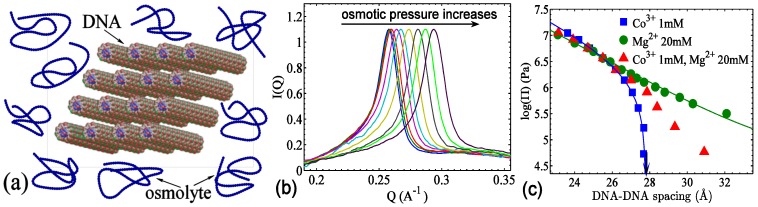
(**a**) Illustration of DNA arrays under osmotic stress; (**b**) Demonstration of shifts of the DNA–DNA X-ray diffraction peak as the osmotic pressure increases under DNA-condensing conditions. The peaks are scaled to assist visualization; (**c**) The force-spacing curves of dsDNA in varied salts as annotated in the legend. The *x*-axis shows the inter-axial spacing, whereas the surface-to-surface spacing is <2 nm. Symbols are experimental data and lines are the fits using exponential forces, as described in the text. The arrow indicates the DNA spacings at zero osmotic pressure.

While its essential role is widely acknowledged, water is more often than not taken for granted as the universal matrix biomolecules live in. Being a polar solvent, water simply acts as a dielectric medium to attenuate electric fields, and it may seem only necessary to introduce minor corrections for likely non-ideal dielectric breakdowns in strong fields. However, in the tight interstitial spaces of thickness ≤ 1 nm, its molecular origin can no longer be neglected. Through a series of pioneering osmotic stress measurements and codifications, Parsegian, Rau, and co-workers elucidated that water/hydration dominates the magnitude and form of DNA–DNA forces in the last nm of surface separation [120,121,122] (see Figure 10 for illustration of osmotic stress measurements and representative DNA force-extension curves). Specifically, it was observed that, upon the approaching of opposing surfaces, the DNA–DNA force–distance relations show a universal exponential form with a decay length of ~0.3 nm independent of the ionic species or concentration. More generally, such universal behavior for dsDNA forces was shown to hold for a large variety of molecular surfaces, charged (e.g., collagen and DNA) or non-charged (e.g., polysaccharides), cylindrical (e.g., DNA) or planar (e.g., lipid bilayers) [123,124]. This evidence culminates with the establishment of universal hydration forces for charged and polar molecular surfaces. Note that charged surfaces additionally make electrostatic contributions to the force, but the electrostatic interaction is dominated by hydration forces at close surface–surface separations.

Hydration forces can be either repulsive or attractive, in ways similar to “like charges repel and opposite charges attract” [123]. As the hydration shell is polar and can be viewed as pointing away or towards the molecular surface depending on the surface charge, the directionality of the hydration shell causes the approaching surfaces to have either conflicting or conforming hydration structures. Conflicting hydration structures lead to destructive dehydration and consequently repulsive hydration force, while conforming hydration structures lead to attractive hydration force. Hydration forces are generally reported in terms of the osmotic pressure $\text{Π}\left(d\right)=f_{r}e^{-d/{\text{λ}}_{r}}-f_{a}e^{-d/{\text{λ}}_{a}}$, where *d* is the inter-axial distance or surface separation, *f_r_* and *f_a_* are amplitudes of repulsive and attractive forces respectively, and λ*_a_* and λ*_r_* are the decay lengths. Figure 10 briefly illustrates the osmotic stress measurements and representative data in different salt conditions. In the case of counterion-mediated DNA–DNA forces in the last nm of surface separation, the absence of counterions would lead to two similarly homogenously hydrated surfaces and these two “like hydration” structures will collide and give rise to repulsive hydration forces. Addition of monovalent counterions weakens hydration but does not change the directionality of hydration, consequently decreasing the repulsive hydration force. However, for multivalent counterions, they can reverse the charge and hydration locally and create undulating hydration structures of both directions. This results in attractive hydration forces between opposing surfaces, noting that the hydration repulsion term still remains due to imperfect matches and/or incomplete charge neutralization near the surfaces. Divalent cations present an interesting border case for hydration forces because they can reverse the charge locally if confined to a single phosphate group. However, no attractive DNA–DNA hydration force has been observed for non-specifically interacting divalent counterions. It is likely that a divalent counterion is localized in between two phosphate groups and no inversion of the hydration direction is incurred.

### 3.3. The Role of DNA’s Structure

The double helical structure of dsDNA is a critical element of several proposed models of DNA–DNA interactions. One possible role is providing discrete charge groups, regularly spaced charge patterns, and groove structures for counterion habitation. Varying DNA structure thus offers an avenue to test different theoretical models. However, studies of non-B-form DNA structures are relatively few [125]. One example of recent studies is the report of divalent counterion mediated attraction between triple-strand DNA (tsDNA) [92]. tsDNA is more highly charged than dsDNA, with ~40% larger linear charge density and ~32% higher surface charge density. tsDNA also has more regularly spaced charges and has narrower grooves. While shedding new light on the physical origin of DNA condensation, the observation fails, however, to differentiate between existing theoretical models, partly due to the dominant effect of substantially higher charge densities. More recently, it was shown that dsRNA of A-form helical structure is more resistant to condensation than B-form dsDNA [126], strongly suggesting that helical structures play non-negligible roles in nucleic acid interactions. Nonetheless, it is difficult to attribute the difference to a unique structural feature, e.g., counterion penetration toward central axis [127] or less evenly spaced helical charges [128]. This is further complicated by the additional hydroxyl group of RNA compared to DNA.

Electrostatic and hydration forces between DNA helices have proven to be fertile ground for biophysical approaches. The intricate interplay between the participating ions, solvent, and DNA makes it difficult to dissect the individual role of each component. It is also likely that different physical mechanisms may be invoked for DNA–DNA interactions in different settings. In order to parse the roles of ions, solvent, and DNA structures, systematic studies of the pertinent variables will be needed. Their strong interdependence further calls for new experimental probes and new theoretical treatments. While DNA–DNA forces can be measured, little experimental knowledge exists of the interstitial space filled with ions and solvent. Important unanswered questions include the ion hydration and distribution, DNA hydration structure, and how the interstitial space varies with ionic condition and DNA structure. Experimentally, the ability to directly probe ions and solvents will be very useful, as exemplified by a recent study to measure the number of ions within dsDNA arrays [102]. However, given the likely amorphous nature of the interstitial ions and solvent, inclusive theoretical models are probably better positioned to capture the relevant physics of nucleic acid interactions. An important step towards this direction was reported by He and Chen [129], including ion–ion correlation and dynamics, solvent polarization, and atomic DNA structure in a consistent framework. It was shown that ion-induced restructuring of the solvent polarization contributes significantly to DNA–DNA interactions. Further developments of such theoretical models will be essential to elucidate the intricate interplays between ions, solvent, and DNA structure.

## 4. Conclusions

The importance of mechanics, electrostatics, and hydration reveals the fundamental DNA molecular forces. Advances in instrumentation allow us to explore the interaction forces in detail and to further predict the behavior of DNA, which will benefit both our understanding of living systems and the creation of smart biomaterials. The DNA molecular force is a key determinant in the operation of cellular machinery in living organisms. An example is the tightly packaged DNA in bacteriophages, where both the mechanical and the electrostatic forces of DNA work together to create a large internal pressure that propel the initial DNA translocation when infecting bacteria. Outside the living system, the quantitative force information of DNA should help us to better design systems that apply DNA in nanotechnologies such as programmable self-assembly, DNA computing, material assembly, and nanomedicine.
